# Valbenazine improves quality of life, functionality, and movement severity in patients with tardive dyskinesia: findings from the KINECT-PRO™ study

**DOI:** 10.1017/S1092852926101138

**Published:** 2026-08-11

**Authors:** Christoph U. Correll, M. Mercedes Perez-Rodriguez, Joseph P. McEvoy, David V. Sheehan, Gustavo Alva, Susan Mathias, Morgan Bron, Robert H. Farber, Hui Zhang, Eduardo Dunayevich, Ashok Parameswaran

**Affiliations:** 1 Department of Psychiatry Research, The Zucker Hillside Hospital, Glen Oaks, NY, USA; 2 Department of Psychiatry and Molecular Medicine, The Donald and Barbara Zucker School of Medicine at Hofstra/Northwell, Hempstead, NY, USA; 3 Department of Child and Adolescent Psychiatry, Charité - Universitätsmedizin Berlin, Berlin, Germany; 4 German Center for Mental Health (DZPG), Partner Site Berlin, Berlin, Germany; 5 Department of Psychiatry, Icahn School of Medicine at Mount Sinai, New York, NY, USA; 6 Augusta University Medical College of Georgia, Augusta, GA, USA; 7 University of South Florida Morsani College of Medicine, Tampa, FL, USA; 8 University of California Riverside Medical School, Riverside, CA, USA; 9 Hoag Hospital, Newport Beach, CA, USA; 10 ATP Clinical Research, Costa Mesa, CA, USA; 11 Health Outcomes Solutions, Palm Beach Gardens, FL, USA; 12 Neurocrine Biosciences Inc., San Diego, CA, USA; 13 Neurona Therapeutics Inc., South San Francisco, USA

**Keywords:** Tardive dyskinesia, patient-reported outcomes, quality of life, functionality, VMAT2, valbenazine

## Abstract

**Background/Objective:**

Once-daily INGREZZA® (valbenazine), a selective vesicular monoamine transporter 2 (VMAT2) inhibitor, is approved for tardive dyskinesia (TD) and Huntington’s chorea. KINECT-PRO™ (NCT05859698) is currently the only study to assess the effects of a VMAT2 inhibitor (valbenazine) on quality of life (QoL) and functionality in patients with TD using multiple validated patient-reported outcomes (PROs).

**Methods:**

Adults with mild-to-severe TD movement severity (per Abnormal Involuntary Movement Scale [AIMS] item 8) and awareness of TD with mild-to-severe associated distress (per AIMS item 10) received open-label valbenazine (40, 60, or 80 mg) for 24 weeks. Primary endpoints included changes from baseline (CFBs) at Week 24 in 3 validated QoL/functionality PROs: Tardive Dyskinesia Impact Scale (TDIS), the only psychometrically validated PRO developed specifically for TD; Sheehan Disability Scale (SDS); and EuroQoL Group’s EQ-Visual Analogue Scale (EQ-VAS). Secondary endpoints included CFB in AIMS total score.

**Results:**

Substantial and clinically meaningful improvements in patient-reported QoL/functionality and clinician-reported TD severity were observed throughout treatment with valbenazine in the overall study population and regardless of psychiatric diagnosis (mood disorders, psychotic disorders) or TD severity at baseline (mild, moderate/severe). At Week 24 (*N* = 45), mean CFBs were: TDIS (−8.0); SDS Social/Leisure (−2.3); SDS Family/Home (−1.6); EQ-VAS (+13.1); AIMS (−6.8). Previously established minimal clinically important difference (MCID) thresholds for TD were exceeded for TDIS and AIMS. Safety and tolerability were consistent with valbenazine’s known profile.

**Conclusions:**

KINECT-PRO is the first study of its kind, and valbenazine is the only VMAT2 inhibitor to demonstrate QoL/functional improvements in patients with TD across multiple validated PROs.

## Introduction

Tardive dyskinesia (TD) is a potentially debilitating movement disorder associated with prolonged exposure to antipsychotics and other dopamine-receptor blocking agents (DRBAs).^1^ ^,^^2^ Antipsychotic medications are the primary treatment for psychotic disorders, and many are FDA-approved as monotherapies or adjunctive therapies for other indications. These conditions include bipolar I and II disorders, major depressive disorder, irritability associated with autistic disorder, Tourette’s syndrome, and agitation related to Alzheimer’s dementia. Because all antipsychotic medications (including second-generation antipsychotics) have a risk of causing TD, the rising on-label and off-label use of these agents necessitates greater awareness of appropriate TD screening and treatment. All individuals taking any antipsychotic medication or other DRBAs should be screened regularly for TD.^3^ ^,^^4^ Regardless of severity, TD can substantially diminish a patient’s quality of life (QoL). Clinical guidelines recommend the use of a vesicular monoamine transporter 2 (VMAT2) inhibitor as first-line treatment for TD, even in individuals whose movement severity is considered mild but impactful.^5^

Two VMAT2 inhibitors, INGREZZA® (valbenazine) and AUSTEDO® (deutetrabenazine), are currently approved in the US for the treatment of TD.^6^ ^,^^7^ Primary evidence for the efficacy of these medications is based on improvements in the severity of TD movements, as assessed using the clinician-rated Abnormal Involuntary Movement Scale (AIMS).^8^ The AIMS, which was developed to evaluate the effects of psychopharmacologic interventions on abnormal movements, has been used as an assessment tool in clinical practice and as a primary endpoint in TD clinical trials.^9^ ^–^^12^

The AIMS includes assessments of movement severity across 7 body regions (items 1–7), along with global clinical judgments for the following: movement severity (item 8, based on the impression of overall severity or the highest single score on items 1–7); incapacitation due to abnormal movements (item 9); and the patient’s awareness of their abnormal movements and level of associated distress (item 10). However, the clinician-rated AIMS does not provide insights into QoL and functionality from a patient perspective, and TD is known to have negative physical, social, and emotional impacts on patients’ lives.^13^ ^–^^17^ Even among patients who have mild abnormal movements per clinician assessment, TD can negatively affect physical functioning (eg, difficulty with swallowing, breathing, walking), have emotional and social consequences (eg, embarrassment, stigmatization of their psychiatric condition, withdrawal from social activities), contribute to impairments (eg, inability to work or attend school), and diminish an individual’s overall health.

To date, no study has been published with the primary goal of assessing the effects of a VMAT2 inhibitor on QoL and functionality in patients with TD. To address this gap, the KINECT-PRO™ (NCT05859698) clinical trial of valbenazine had 2 objectives: first, to evaluate patient-reported changes in TD impact, functional impairment (social, family, work), and overall health; and second, to evaluate clinician-reported and patient-reported changes in TD severity.

## Methods

### Study design, participants, and treatment

KINECT-PRO was a Phase 4 study, conducted from May 2023 to December 2024. Participants were screened at 24 study centers in the US and enrolled at 18 centers. The study adhered to International Council for Harmonisation Guidelines for Good Clinical Practice, US Code of Federal Regulations standards, and FDA guidelines. The study protocol, informed consent forms, and other study documents were approved by an institutional review board (Advarra, Inc., Columbia, MD). All participants provided informed and written consent before any study procedure was initiated.

Study participants were outpatients aged ≥18 years who had a TD diagnosis (per *Diagnostic and Statistical Manual of Mental Disorders, 5^th^ Edition* [DSM-5] criteria^18^) for ≥3 months before screening. Other key inclusion criteria included: confirmed diagnosis of schizophrenia, schizoaffective disorder, bipolar disorder, or major depressive disorder as defined by the DSM-5; meeting the Schooler-Kane criteria for TD, defined as at least moderate abnormal movements in at least 1 body region (ie, score ≥3 in any of the first 7 AIMS items) or at least mild in at least 2 body regions (ie, score ≥2 in ≥2 of the first 7 AIMS items)^19^; mild-to-severe TD movements, defined as a score ≥2 on item 8 of the AIMS; and awareness of dyskinetic movement with at least mild associated distress, defined as a score ≥2 on item 10 of the AIMS. Full inclusion/exclusion criteria are listed in Supplementary Table S1.

**Figure 1.**
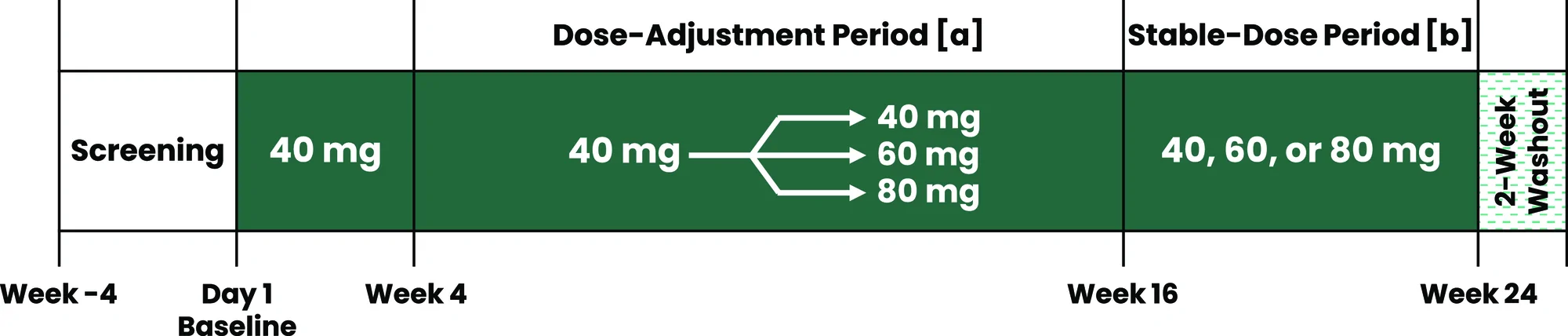
Study design. In the dose-adjustment period [a], increases were allowed up to the individual’s highest tolerable dose (max: 80 mg) or decreased for safety or tolerability. In the stable-dose period [b], no changes were strongly encouraged; however, decreases in dosing were allowed as needed for safety or tolerability.

KINECT-PRO included a screening period (up to 4 weeks, with extension up to 2 weeks for unavoidable circumstances), an open-label treatment period (24 weeks), and a safety follow-up period (2 weeks) (Figure 1). From baseline (Day 1) through end of Week 4, participants received valbenazine at 40 mg, the lowest clinically effective dose. From end of Week 4 through Week 16, the valbenazine dose could be adjusted to 60 or 80 mg based on individual treatment needs, investigators’ clinical judgment, and/or tolerability. From the end of Week 16 through Week 24, stable dosing was strongly encouraged; however, dose decreases were allowed for safety or tolerability. Valbenazine was administered in all participants in accordance with the approved prescribing information, including use of the 40 mg dose in participants who had moderate or severe hepatic impairment, were known CYP2D6 poor metabolizers, or were taking a strong CYP3A4 or CYP2D6 inhibitor (due to increased exposure to valbenazine and/or its active metabolite).^6^ Allowed and prohibited concomitant medications are summarized in Supplementary Table S2. Dosing of allowed concomitant medications was expected to remain stable throughout the study.

### Efficacy assessments

Changes from baseline (CFBs) at Week 24 in the following PROs were defined as primary endpoints: Tardive Dyskinesia Impact Scale (TDIS),^20^ Sheehan Disability Scale (SDS),^21^ and the EuroQoL Group’s EQ-visual analogue scale (EQ-VAS).^22^ ^,^^23^ The TDIS measures the physical, social, and emotional impacts of TD, and it is the first and only psychometrically validated PRO specific for TD. It consists of 11 items organized into 6 conceptual groupings: mouth/throat function, dexterity, mobility, pain, social, and emotional. For each TDIS item, patients rated their impact over the “past 7 days including today” from 0 (impacted “not at all” or “never”) to 4 (impacted “extremely” or “all the time”). Change from baseline in the summed total score (range, 0 to 44) was used for the endpoint. The SDS measures disability and impairment in 3 critical domains of functioning, with items related to work/school work (“Work/School”), social life/leisure activities (“Social/Leisure”), and family life/home responsibilities (“Family/Home”). For each of these items, patients rated the level of disruption due to symptoms from 0 (“not at all”) to 10 (“extremely”). The EQ-VAS is a single item measuring current health status used to evaluate health-related quality of life (HRQoL), with scores ranging from 0 (“worst health you can imagine”) to 100 (“best health you can imagine”).

Secondary endpoints included CFBs at Week 24 in the AIMS total score (sum of items 1–7) and the Clinician Global Impression of Severity–Tardive Dyskinesia (CGI-TD-S) score. Items 1–7 of the AIMS measure movement severity across body regions, with each item scored from 0 (“none, normal”) to 4 (“severe”) and the total score ranging from 0 to 28.^8^ The CGI-TD-S is a single item with scores ranging from 1 (“normal, not at all ill”) to 7 (“among the most severely ill patients”).^8^ Changes in TD severity were also evaluated using the Patient Global Impression of Change (PGI-C), a single-item PRO with scores ranging from 1 (“very much improved”) to 7 (“very much worse”) from initiation of the study.^8^ The percentage of participants who had a PGI-C score ≤ 2 (“much improved” or “very much improved”) at Week 24 was defined as a secondary endpoint.

The percentage of participants who met a threshold for TD symptomatic remission at Week 24 was also analyzed in an exploratory post hoc analysis. This threshold, derived from the Schooler-Kane criteria for TD^19^ and proposed by Kane et al.,^24^ defines symptomatic remission as having a movement severity score of 0 (“none”) or 1 (“minimal, may be extreme normal”) in each of the 7 body regions of the AIMS (items 1–7).

### Sample size considerations

For this open-label single-arm study, it was assumed that 74% of participants would complete the Week 24 assessments. Therefore, enrollment was targeted at approximately 60 participants so that 44 participants would be included in the primary analyses. To ensure adequate representation of different backgrounds, the study aimed to enroll similar numbers of participants in subgroups that were defined by psychiatric diagnosis (psychotic disorders, mood disorders) and TD severity at baseline (mild, moderate-to-severe).

### Efficacy analyses

Efficacy outcomes were analyzed in the full analysis set (FAS), defined as all participants who had data available at baseline and at ≥1 post-baseline visit. All primary and secondary assessments (TDIS, SDS, EQ-VAS, AIMS, CGI-TD-S, and PGI-C) were also analyzed in subgroups of participants characterized by psychiatric diagnosis (schizophrenia or schizoaffective disorder [“Psychotic”], bipolar disorder or major depressive disorder [“Mood”]) and by TD movement severity per AIMS item 8 at baseline (score of 2 [“TD-Mild”], score of 3 or 4 [“TD-Mod/Severe”]).

Descriptive statistical methods were used to summarize study data. Mean values at baseline and Week 24 are presented with standard deviations (SDs). Mean CFBs are presented with 95% confidence intervals (95% CIs). No confirmatory testing or calculation of p-values was performed as this was a single-arm study. Percentages are presented with n-values (eg, number of participants meeting the PGI-C response threshold). All analyses were based on observed values, with no imputation for missing data or adjustments for multiplicity. No statistical comparisons between subgroups were performed.

### Safety assessments and analyses

Safety outcomes were analyzed descriptively in the safety analysis set, defined as all participants who took ≥1 dose of study treatment. Safety assessments included treatment-emergent adverse events (TEAEs), clinical laboratory tests, vital signs, electrocardiograms, Columbia-Suicide Severity Rating Scale (C-SSRS), Barnes Akathisia Rating Scale (BARS), and modified Simpson-Angus Scale (mSAS).

## Results

### Study participants

**Figure 2.**
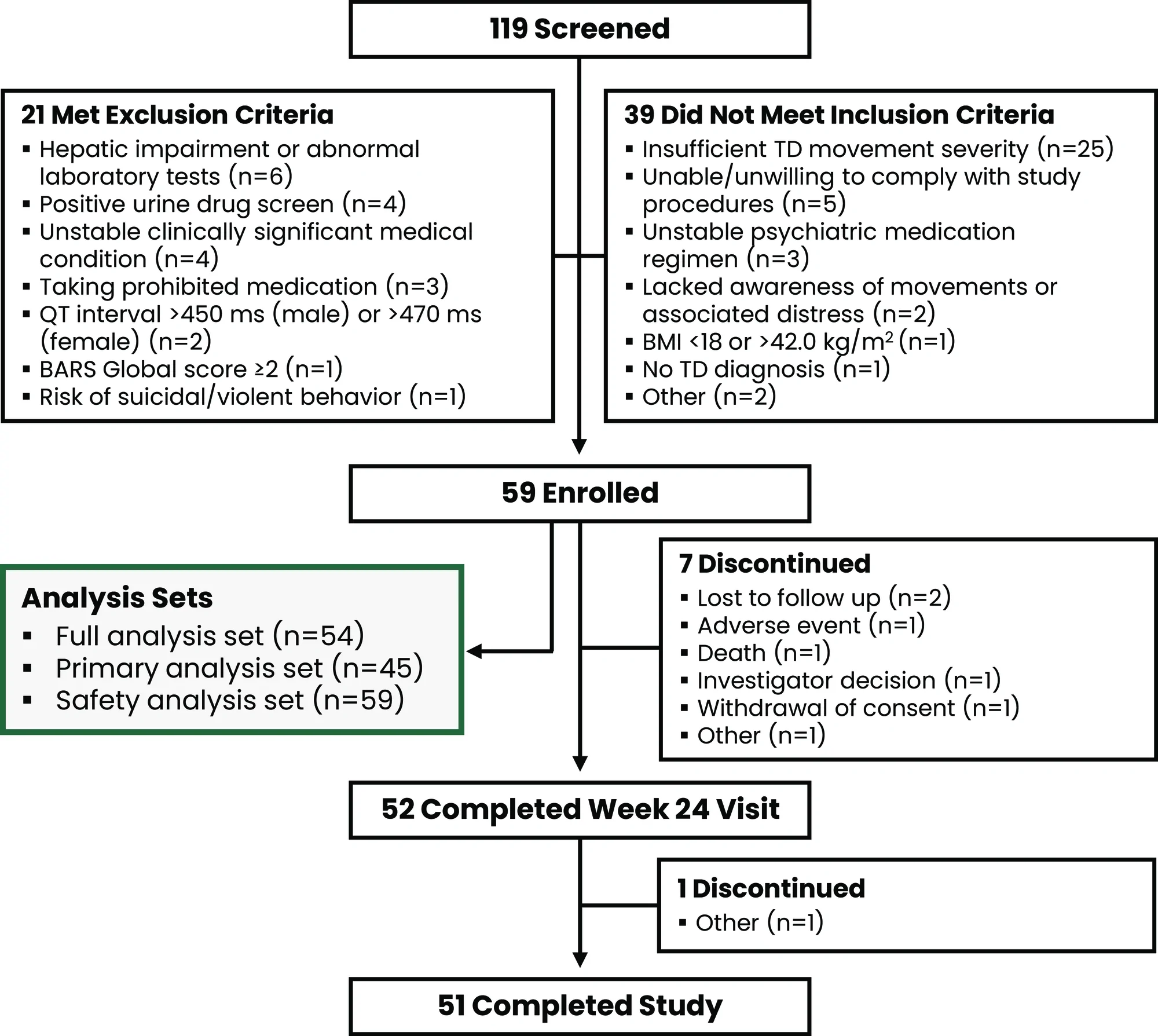
Study flow. The full analysis set (FAS) included all participants with an efficacy assessment at baseline and at ≥1 post-baseline visit. The primary analysis set included all participants in the FAS who had an available efficacy assessment at baseline and Week 24. Due to the small number of participants who were working or attending school, 10 participants were included in the analysis for SDS Work/School. The safety analysis set included all participants who received ≥1 dose of study treatment. *Note:* BARS, Barnes Akathisia Rating Scale; BMI, body mass index; TD, tardive dyskinesia. Figure 2. long description.

A total of 59 participants were enrolled in the study; their demographic and clinical characteristics are shown in Table 1. Of these 59 enrolled participants, 52 (88.1%) completed treatment (Week 24) and 51 (86.4%) completed the study through the end of the safety follow-up period (Figure 2).

**Table 1.** Demographics and Baseline Characteristics Table 1. long description.

	All participants (*N* = 59)
Age, mean (SD), years	61.3 (12.0)
Sex, *n* (%)	
Female	34 (57.6)
Male	25 (42.4)
Race, *n* (%)	
White	38 (64.4)
Black/African American	17 (28.8)
Other/multiple^a^	4 (6.8)
Ethnicity, *n* (%)	
Hispanic or Latino	24 (40.7)
Not Hispanic or Latino	35 (59.3)
Body mass index, mean (SD), kg/m^2^	29.9 (6.4)
Baseline TD severity, *n* (%)	
Mild: AIMS item 8 score = 2	24 (40.7)
Moderate/severe: AIMS item 8 score = 3 or 4	35 (59.3)
Psychiatric diagnosis, *n* (%)	
Schizophrenia or schizoaffective disorder	27 (45.8)
Major depressive disorder or bipolar disorder	32 (54.2)
Baseline PRO scores, mean (SD)^b^ ^,^^c^	
Tardive Dyskinesia Impact Scale total, range 0 to 44	14.3 (8.1)
Sheehan Disability Scale domains, range 0 to 10	
Work/school^d^	3.1 (3.2)
Social life/leisure activities	4.4 (2.8)
Family life/home responsibilities	3.2 (3.0)
EQ-Visual Analogue Scale, range 0 to 100	66.1 (18.0)
Baseline ClinRO scores, mean (SD)^b^ ^,^^c^	
Abnormal Involuntary Movement Scale total, range 0 to 28	11.1 (4.0)
Clinical Global Impression of Severity–Tardive Dyskinesia, range 1 to 7	3.9 (0.7)

Abbreviation: ClinRO, clinician-rated outcome; PRO, patient-reported outcome; TD, tardive dyskinesia; SD, standard deviation.

a Asian (*n* = 1); other, not specified (*n* = 2); multiple, not specified (*n* = 1).

b Baseline scores analyzed in the full analysis set (*n* = 54).

c Higher scores indicate worse impact (TDIS), disruption/impairment (SDS), movement severity (AIMS), and overall severity (CGI-TD-S); lower score indicates worse health state (EQ-VAS).

d Scores analyzed in participants with data at baseline (*n* = 17).

After completion of initial dosing with valbenazine 40 mg at the end of Week 4 (*n* = 53), 16 (30.2%) participants continued with 40 mg, 34 (64.2%) had a dose increase to 60 mg, and 3 (5.7%) increased to 80 mg. Of participants who were receiving valbenazine treatment at the end of Week 24 (*n* = 50), 13 (26.0%) were taking valbenazine 40 mg, 12 (24.0%) were taking 60 mg, and 25 (50.0%) were taking 80 mg. An analysis of each participant’s dosing over time indicated the following: most who had no dose increase at Week 4 remained on valbenazine 40 mg through Week 24; and most with an increase to 60 or 80 mg during the 12-week dose-adjustment period remained on those doses through Week 24 (Supplementary Figure S1). No dose reductions occurred during the stable-dose period (end of Week 16 through Week 24), except for 1 participant who tried 80 mg and was reduced back to 60 mg after a few days.

### Effects of valbenazine on patient-reported quality of life and functionality

**Figure 3.**
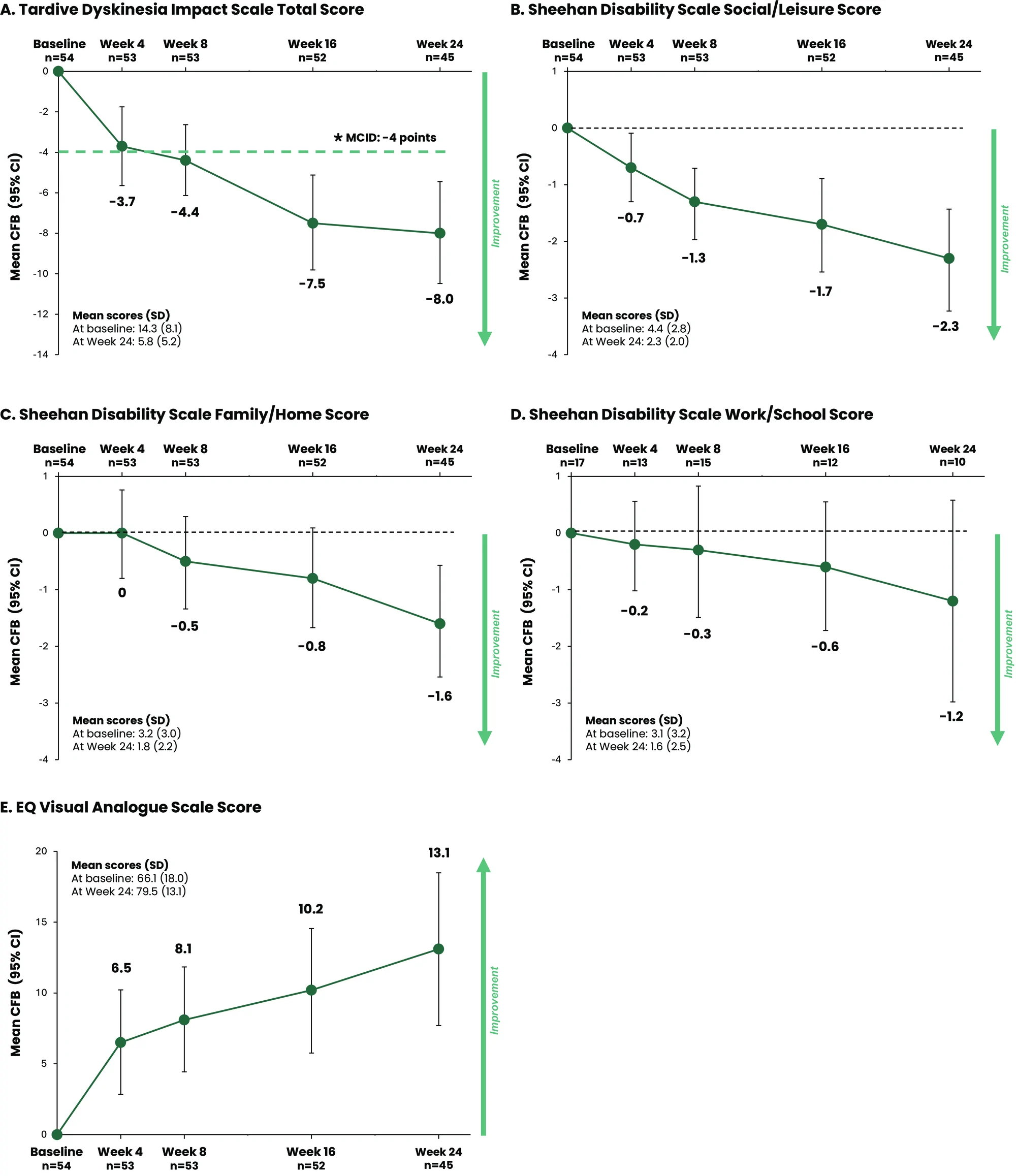
Quality-of-life/functionality outcomes (FAS). *Note:* *MCID from Bron M, et al. *J Clin Psychiatry.* 2026;87(2):25nr16047. CFB, change from baseline; CI, confidence interval; EQ, EuroQoL Group; FAS, full analysis set; MCID, minimal clinically important difference; SD, standard deviation. Figure 3. long description.

The FAS included 54 participants, 45 (83.3%) of whom had available data at Week 24 for all primary assessments, except for the SDS Work/School domain because few participants reported having worked or attended school in the past week (*n* = 17 at baseline, *n* = 10 with available score change data at Week 24). Mean improvements in primary endpoints were observed throughout treatment, with improvements generally increasing over time (Figure 3). At Week 24, mean CFBs [with 95% CIs] were as follows for the QoL/functionality PROs: TDIS total score, −8.0 [−10.5, −5.4]; SDS Work/School, −1.2 [−3.0, +0.6]; SDS Social/Leisure, −2.3 [−3.2, −1.4]; SDS Family/Home, −1.6 [−2.5, −0.6]; and EQ-VAS, +13.1 [+7.7, +18.5].

In TD, a minimal clinically important difference (MCID) threshold has been previously established for the TDIS (−4 points).^25^ In this study, mean changes in TDIS total score exceeded this MCID threshold from Week 8 (first visit after dose escalation was allowed) through Week 24 of valbenazine treatment (Figure 3a). Confidence intervals are presented to describe the precision of the estimates. Interpretation remains descriptive and exploratory due to the single-arm design of the study.

### Effects of valbenazine on TD severity

**Figure 4.**
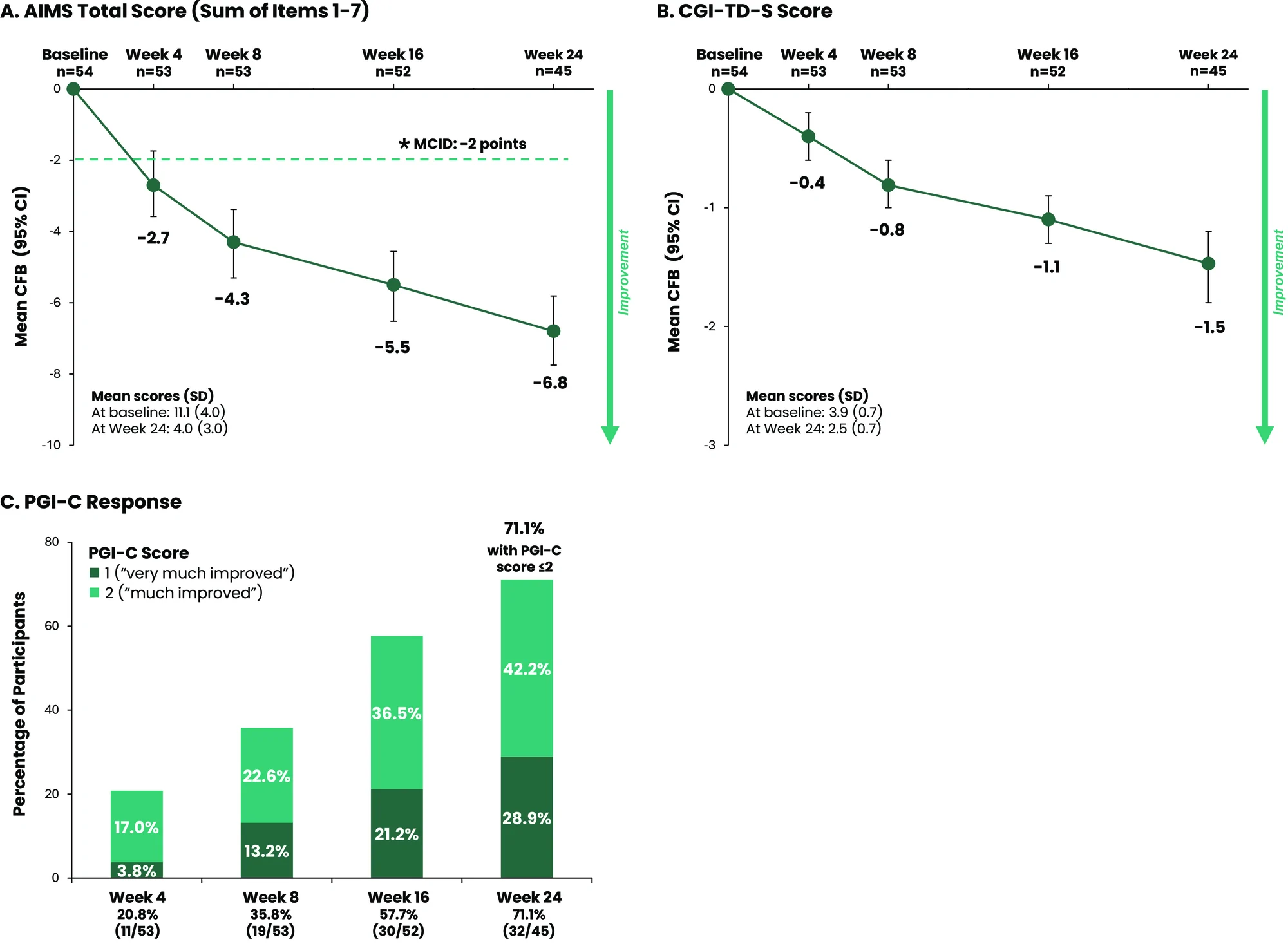
TD movement severity outcomes (FAS). *Note:* *MCID from Stacy M, Sajatovic M, Kane JM, et al. *Mov Disord.* 2019;34(8):1203–1209. AIMS, Abnormal Involuntary Movement Scale, CFB, change from baseline; CGI-TD-S, Clinical Global Impression of Severity–Tardive Dyskinesia; CI, confidence interval; FAS, full analysis set; PGI-C, Patient Global Impression of Change; SD, standard deviation; TD, tardive dyskinesia. Figure 4. long description.

Mean improvements in the secondary assessments were also observed throughout treatment (Figure 4). At Week 24 (*n* = 45), mean CFBs [with 95% CIs] in the clinician-reported outcomes (ClinROs) were as follows: AIMS (−6.8 [−7.8, −5.8]); CGI-TD-S (−1.5 [−1.8, −1.2]). The threshold for TD symptomatic remission was met by 57.8% (26/45) of participants at Week 24. For the PGI-C, 71.1% (32/45) of participants had a score ≤ 2 (“much improved” or “very much improved”) at Week 24. Mean changes in AIMS total score exceeded the MCID (−2 points^26^) at all post-baseline visits (Figure 4a).

### Subgroup analyses

Patient-reported QoL and functionality improved in all 4 subgroups (Table 2). Mean scores at baseline indicated substantial negative impact and functional impairment in all 4 subgroups, including participants who had mild TD movement severity per clinician assessment. The impacts were especially prominent in the Mood subgroup, potentially reflecting greater insights into their movements as compared to patients with schizophrenia. All QoL PROs (TDIS, SDS, EQ-VAS) improved substantially from baseline through Week 24, regardless of psychiatric diagnosis or severity of TD movements at baseline. At Week 24, all 4 subgroups demonstrated clinically meaningful improvements in TD impacts (TDIS score reduction of ≥4 points) and TD movement severity (AIMS score reduction of ≥2 points) (Table 3). Across subgroups, 65.0% to 76.0% of participants rated their TD symptoms as “much improved” or “very much improved” (ie, PGI-C score ≤2) since starting valbenazine treatment.

**Table 2.** Quality-of-Life Outcomes by Subgroup^a^ Table 2. long description.

	At baseline		At Week 24		At Week 24	
	n	Mean score (SD)	n	Mean score (SD)	n	Mean CFB [95% CI]
TDIS total						
Mood	30	15.9 (6.8)	25	5.1 (5.0)	25	−9.7 [−12.7, −6.7]
Psychotic	24	12.4 (9.3)	20	6.7 (5.5)	20	−5.8 [−10.2, −1.4]
TD-Mild	22	12.9 (8.0)	20	6.2 (4.7)	20	−6.8 [−10.7, −2.9]
TD-Mod/Severe	32	15.3 (8.1)	25	5.6 (5.7)	25	−8.9 [−12.4, −5.4]
SDS social/leisure^b^						
Mood	30	4.7 (2.5)	25	2.0 (1.6)	25	−2.9 [−4.1, −1.7]
Psychotic	24	4.1 (3.1)	20	2.7 (2.4)	20	−1.6 [−3.0, −0.2]
TD-Mild	22	3.9 (2.6)	20	2.2 (2.2)	20	−1.8 [−3.3, −0.2]
TD-Mod/Severe	32	4.8 (2.9)	25	2.4 (1.9)	25	−2.8 [−3.9, −1.7]
SDS family/home^b^						
Mood	30	3.8 (2.6)	25	1.6 (1.8)	25	−2.3 [−3.5, −1.0]
Psychotic	24	2.5 (3.2)	20	2.2 (2.6)	20	−0.7 [−2.3, +1.0]
TD-Mild	22	2.9 (2.8)	20	1.9 (2.1)	20	−1.3 [−3.0, +0.4]
TD-Mod/Severe	32	3.4 (3.1)	25	1.8 (2.3)	25	−1.8 [−3.0, −0.5]
EQ-VAS						
Mood	30	62.6 (16.5)	25	80.1 (14.2)	25	+17.0 [+9.3, +24.7]
Psychotic	24	70.4 (19.2)	20	78.8 (11.9)	20	+8.3 [+0.7, +15.8]
TD-Mild	22	65.6 (15.7)	20	80.0 (12.4)	20	+12.8 [+4.0, +21.7]
TD-Mod/Severe	32	66.4 (19.6)	25	79.1 (13.8)	25	+13.3 [+6.1, +20.6]

Abbreviation: AIMS, Abnormal Involuntary Movement Scale; CFB, change from baseline; CI, confidence interval; EQ‑VAS, EuroQoL Group’s EQ-Visual Analogue Scale; Mood, bipolar disorder or major depressive disorder; Psychotic, schizophrenia or schizoaffective disorder; SD, standard deviation; SDS, Sheehan Disability Scale; TD, tardive dyskinesia; TDIS, Tardive Dyskinesia Impact Scale.

a Subgroups are presented by psychiatric diagnosis (Mood, Psychotic) and by TD severity at baseline (TD‑Mild [AIMS item 8 score of 2], TD-Mod/severe [AIMS item 8 score of 3 or 4]).

b Data for SDS Work/School are not shown due to the small number of participants with available data at baseline (Mood [*n* = 12], Psychotic [*n* = 5], TD-Mild [*n* = 6], TD-Mod/severe [*n* = 11]) and change from baseline at week 24 (Mood [*n* = 5], Psychotic [*n* = 5], TD-Mild [*n* = 4], TD-Mod/severe [*n* = 6]).

**Table 3.** TD Movement Severity Outcomes by Subgroup^a^ Table 3. long description.

	At baseline		At Week 24		At Week 24	
AIMS total	*n*	Mean score (SD)	*n*	Mean score (SD)	*n*	Mean CFB [95% CI]
Mood	30	12.0 (4.1)	25	4.0 (3.3)	25	−7.6 [−8.9, −6.3]
Psychotic	24	10.0 (3.7)	20	3.9 (2.6)	20	−5.8 [−7.1, −4.4]
TD-Mild	22	9.1 (3.3)	20	3.4 (3.1)	20	−5.6 [−7.0, −4.1]
TD-Mod/Severe	32	12.5 (3.9)	25	4.4 (2.8)	25	−7.8 [−9.0, −6.5]
CGI-TD-S	*n*	Mean score (SD)	*n*	Mean score (SD)	*n*	Mean CFB [95% CI]
Mood	30	4.1 (0.7)	25	2.6 (0.7)	25	−1.5 [−1.9, −1.1]
Psychotic	24	3.8 (0.8)	20	2.4 (0.8)	20	−1.4 [−1.9, −0.9]
TD-Mild	22	3.5 (0.6)	20	2.3 (0.8)	20	−1.2 [−1.7, −0.7]
TD-Mod/Severe	32	4.3 (0.6)	25	2.7 (0.6)	25	−1.7 [−2.0, −1.3]
PGI-C^b^	--	--	--	--	*n*	With score ≤ 2, n (%)
Mood	--	--	--	--	25	19 (76.0)
Psychotic	--	--	--	--	20	13 (65.0)
TD-Mild	--	--	--	--	20	14 (70.0)
TD-Mod/Severe	--	--	--	--	25	18 (72.0)

Abbreviation: AIMS, Abnormal Involuntary Movement Scale; CFB, change from baseline; CGI-TD-S, Clinical Global Impression of Severity–Tardive Dyskinesia; CI, confidence interval; EQ‑VAS, EuroQoL Group’s EQ-Visual Analogue Scale; Mood, bipolar disorder or major depressive disorder; Psychotic, schizophrenia or schizoaffective disorder; SD, standard deviation; SDS, Sheehan Disability Scale; TD, tardive dyskinesia; TDIS, Tardive Dyskinesia Impact Scale.

a Subgroups are presented by psychiatric diagnosis (Mood, Psychotic) and by TD severity at baseline (TD‑Mild [AIMS item 8 score of 2], TD-Mod/Severe [AIMS item 8 score of 3 or 4]).

b As the PGI-C is a measure of change, the assessment is not administered at baseline. Therefore, the numbers and percentages of participants with a score ≤ 2 (“much improved” or "very much improved”) at Week 24 are presented in the last column.

### Safety

In the safety analysis set (*N* = 59), 28 (47.5%) participants had ≥ 1 TEAE (Table 4). A total of 55 TEAEs were reported, 47 (85.5%) of which were considered mild or moderate, and 41 (74.5%) of which were judged by the investigator as not related or unlikely to be related to study drug. The three most common TEAEs—nausea, somnolence, and suicidal ideation—each occurred in 3 (5.1%) participants.

**Table 4.** Treatment-Emergent Adverse Events Table 4. long description.

	All participants (*N* = 59)
Summary, *n* (%)	
Any TEAE	28 (47.5)
Any TEAE leading to a dose reduction	2 (3.4)
Any TEAEs leading to study drug discontinuation	2 (3.4)
Any serious TEAE	6 (10.2)
Any TEAE resulting in death^a^	1 (1.7)
Common TEAEs, *n* (%)^b^	
Nausea	3 (5.1)
Somnolence	3 (5.1)
Suicidal ideation	3 (5.1)
Depression	2 (3.4)
Headache	2 (3.4)
Insomnia	2 (3.4)
Tremor	2 (3.4)
Vomiting	2 (3.4)

Abbreviation: TEAE, treatment-emergent adverse event; SD, standard deviation.

a Due to active gastrointestinal bleeding in an 81-year-old female participant with a history of peptic ulcers; judged by the investigator as not related to study drug.

b Reported in ≥2% of participants.

Serious TEAEs were reported in 6 (10.2%) participants. No serious TEAEs occurred in >1 participant and none were judged by the investigator as related to treatment. Two participants with serious TEAEs were discontinued from treatment: a 44-year-old female participant in the Psychotic subgroup with chronic obstructive pulmonary disease; and a 70-year-old male participant in the Mood subgroup with suicidal ideation. These serious TEAEs resolved after drug withdrawal. One death occurred in an 81-year-old female participant in the Psychotic subgroup with a history of peptic ulcers who developed complications from gastrointestinal bleeding. This death was judged by the investigator as not related to study treatment.

Two participants had TEAE(s) that led to dose reductions: a 56-year-old female in the Psychotic subgroup with moderate intermittent nausea and a 69-year-old female in the Mood subgroup who experienced mild nausea, dizziness/loss of balance, difficulty swallowing, and headaches. These TEAEs resolved after the valbenazine dose was reduced, and neither participant was discontinued from treatment.

Among the 59 participants in the safety analysis set, 19 (32.2%) had a lifetime history of suicidal ideation (C-SSRS Category 1–5). Two participants had suicidal ideation at a post-baseline visit: one who expressed a wish to be dead (C-SSRS Category 1); and one who had suicidal ideation with no plan or intent to act (C-SSRS Category 2). One additional participant had a serious TEAE of suicidal ideation, but without any report of C-SSRS ideation.

No clinically meaningful CFBs in clinical laboratory tests, vital signs, electrocardiograms, BARS scores, or mSAS score were observed at Week 24 (Supplementary Table S3).

## Discussion

Approvals of the VMAT2 inhibitors valbenazine and deutetrabenazine for the treatment of TD (in April and August 2017, respectively) were based on their statistical superiority to placebo using the AIMS total score.^9^ ^–^^12^ These studies were not designed to focus on the impact of TD on the participants’ QoL and their physical, social, and emotional functioning. KINECT-PRO is the first trial of a VMAT2 inhibitor (valbenazine) to address this gap by reporting the effects of TD treatment on QoL and functionality, using multiple validated PROs.

The primary objective of KINECT-PRO was to assess the effects of valbenazine on several patient-reported measures of QoL and functionality in adults with diagnosed TD. Although previous studies of valbenazine have implemented PROs as secondary or exploratory endpoints,^27^ ^,^^28^ KINECT-PRO was designed to specifically assess multiple aspects of patient-reported QoL. After 24 weeks of open-label treatment with once-daily valbenazine, study participants demonstrated mean improvements from baseline across all 3 primary PRO endpoints, indicating improvements in TD impacts (TDIS), functional impairment (SDS), and HRQoL (EQ-VAS).

The secondary objective of KINECT-PRO was to assess the effects of valbenazine using 2 ClinROs (AIMS, CGI-TD-S) and a PRO (PGI-C). These secondary endpoints indicated a substantial reduction in TD movement severity based on clinical assessments in 7 body regions, global improvements in severity per investigator judgment, and global improvements per participants’ self-reports. These improvements were observed by the first post-baseline visit at Week 4, when all participants had been taking the initial 40 mg dose. They generally increased through Week 24, when 50.0%, 24.0%, and 26.0% were taking the 80, 60, and 40 mg doses, respectively.

The clinical relevance of KINECT-PRO outcomes is confirmed by the 2 measures with established MCID thresholds in TD: the TDIS, the only psychometrically validated PRO specifically developed to measure the impacts of TD; and the AIMS, the ClinRO on which the approvals of valbenazine and deutetrabenazine were based. Anchor-based MCIDs for both scales (TDIS -4 points^25^; AIMS -2 points^20^) were estimated using data from prior clinical trials of valbenazine in TD. From Week 8 (the first visit after which dose escalation was allowed) to Week 24 in KINECT-PRO, the mean change in TDIS total score (−8.0 points at Week 24) greatly exceeded the MCID threshold for the TDIS (−4 points). Mean CFBs in the AIMS total score also exceeded the MCID (−2 points) at all study visits through Week 24 (−6.8 points). Interpretation of results within the context of these MCIDs indicates meaningful and consistent improvements in TD impact (TDIS) and movement severity (AIMS) in participants who received once-daily valbenazine for 24 weeks.

Clinically relevant thresholds for changes in SDS, EQ-VAS, and CGI-TD-S scores have not been estimated in a TD population. However, consistent improvements were also observed in these measures, with the magnitudes of change in this single-arm study being supported by the 95% CIs for mean changes at Week 24. As with the TDIS and AIMS, the 95% CIs for SDS Social/Leisure (−2.3 [−3.2, −1.4]), SDS Family/Home (−1.6 [−2.5, −0.6]), EQ-VAS (+13.1 [+7.7, +18.5]), and CGI-TD-S (−1.5 [−1.8, −1.2]) did not cross zero, suggesting robust improvements in functional impairment, HRQoL, and clinician-assessed TD severity after starting valbenazine. PGI-C response rates also suggested meaningful improvement, with 71.1% (32/45) of participants reporting their TD as “much improved” or “very much improved” since starting valbenazine. In conjunction with the TDIS and the AIMS, these results are consistent with improvements observed across endpoints in KINECT-PRO. These improvements also align with findings from other valbenazine studies. A real-world chart-extraction study showed improved QoL in patients with TD treated with valbenazine,^29^ while a randomized placebo-controlled withdrawal study showed maintenance of SDS and EQ-VAS improvements in participants who stayed on valbenazine as secondary endpoints.^30^ ^,^^31^ Long-term studies of valbenazine that implemented the AIMS, Clinical Global Impression of Change, and PGI-C demonstrated sustained and clinically meaningful TD improvements with valbenazine.^28^ ^,^^32^ ^,^^33^

Adding to this breadth of evidence are the subgroup analyses from KINECT-PRO. Severe TD might be expected to result in poor QoL, but even those with “mild” TD severity per clinician assessment were substantially impacted by their TD movements at baseline and saw clinically meaningful improvement with valbenazine treatment.

Compared to patients with schizophrenia, those with non-psychotic mood disorders tend to be more aware of (and more affected by) their TD, even when their movements are relatively less noticeable.^34^ It should not be assumed, however, that all patients with schizophrenia or other psychotic conditions are unaware of their movements or do not experience any distress or impacts due to their TD, as evidenced by the results of this and other studies. In RE-KINECT, a prospective study of patients at risk for TD due to antipsychotic exposure, 75.5% who had abnormal involuntary movements consistent with TD (per clinician assessment) were aware of those movements, with no statistical difference between those who had schizophrenia or schizoaffective disorder and those who had a mood disorder.^27^ Moreover, patient-reported impacts on function, QoL, and daily activities were greater in RE-KINECT patients with possible TD (versus the cohort with no abnormal involuntary movements), particularly among those who were aware of their movements.^17^ To recognize the heterogeneity of the patients who develop and are impacted by TD, KINECT-PRO included similarly sized subgroups of participants who were categorized by psychiatric diagnosis (Mood, Psychotic) and movement severity (TD-Mild, TD-Mod/Severe).

Awareness of TD and associated distress (per AIMS item 10) were required for study entry, and no statistical comparisons between subgroups were conducted. Therefore, caution must be taken when interpreting the subgroup results. However, subgroup results generally support the idea that a broad spectrum of patients with TD can benefit from treatment. All 4 subgroups had substantial patient-reported TD impacts at baseline, as measured by the TDIS. As might be expected, the Mood and TD-Mod/Severe subgroups had higher mean TDIS scores at baseline and, therefore, larger mean TDIS improvements at Week 24. However, all subgroups experienced clinically meaningful (ie, ≥4-point) improvements with valbenazine and had similar TDIS scores at Week 24. These results suggest that all participants, regardless of psychiatric diagnosis or TD movement severity at baseline, experienced meaningful improvements with valbenazine. Subgroup analyses for other PROs (SDS Social/Leisure, SDS Family/Home, EQ-VAS), as well as clinician-rated TD movement severity (AIMS score), showed similar patterns with higher baseline scores in the Mood and TD-Mod/Severe subgroups, but substantial improvements across all subgroups resulting in similar scores at Week 24. AIMS improvements in all subgroups also exceeded the −2-point MCID at Week 24. Thus, improvements in QoL or TD movement severity with valbenazine were not restricted to any particular subgroup based on psychiatric diagnosis or TD movement severity at baseline. Consistent with American Psychiatric Association recommendations,^5^ these results emphasize the need to consider movement severity *and* QoL (impact, impairment, disability) when treating TD.

The safety and tolerability of valbenazine in KINECT-PRO were consistent with the known profile of valbenazine.^6^ No new concerns were identified in terms of TEAEs, clinical laboratory tests, vital signs, electrocardiograms, C-SSRS, BARS, or mSAS.

KINECT-PRO implemented open-label treatment with all approved once-daily valbenazine doses, and the dosing patterns in this study were consistent with real-world practice. In the absence of a placebo control or blinded randomization to a specific dose, the results of this study should be interpreted within the context of the known efficacy of valbenazine, as was established in the Phase 2 and Phase 3 clinical trials.^9^ ^,^^10^ ^,^^28^ ^,^^32^ KINECT-PRO participants and investigators might have been subject to expectation bias, as valbenazine is known to be efficacious in TD. However, taking other valbenazine studies into consideration, this potential for bias did not appear to be a concern. The magnitude of PRO and ClinRO improvements in KINECT-PRO is consistent with findings from the placebo-controlled Phase 2 and Phase 3 trials, the long-term rollover study (conducted prior to valbenazine approval),^33^ and the placebo-controlled Phase 4 randomized withdrawal study.^30^ ^,^^31^ The small number of participants with available data for SDS Work/School was a limitation, and more research in a larger set of patients with TD who are working or attending school is warranted. Finally, the open-label design of KINECT-PRO and limited sample size may limit the interpretation of the safety and tolerability results. However, the nature, severity, and causality of the reported TEAEs in the study are consistent with the known safety and tolerability profile of valbenazine, with no new safety concerns identified during the study.

## Conclusions

KINECT-PRO is the first study of its kind, and valbenazine is the only VMAT2 inhibitor with clinical trial data demonstrating QoL and functional improvements in patients with TD across multiple validated PROs, including the only psychometrically validated PRO that is specific for TD (ie, TDIS). KINECT-PRO reinforces the importance of identifying physical and socio-emotional impacts, functional impairments, and HRQoL across a broad spectrum of patients with TD, especially in those with TD that may be considered “mild” in severity. The results of the study indicate clinically meaningful and consistent improvements in QoL/functionality and TD movement severity in participants who received up to 24 weeks of valbenazine, including the subgroup with “mild” TD movement severity. These results contribute to an existing body of evidence that is consistent with established efficacy and safety evidence for once-daily valbenazine in TD. The KINECT-PRO study highlights the importance of treating TD in a more comprehensive way—one in which each patient’s particular physical, social, and emotional needs are considered when developing an appropriate treatment plan.

## Supporting information

10.1017/S1092852926101138.sm001 Correll et al. supplementary material Correll et al. supplementary material

## Supplementary material

The supplementary material for this article can be found at http://doi.org/10.1017/S1092852926101138.

## Long descriptions

### Table 1. Long description

The table contains the following data for All participants (N = 59):* Age: mean (S D) of 61.3 (12.0) years.* Sex: Female 34 (57.6 percent); Male 25 (42.4 percent).* Race: White 38 (64.4 percent); Black or African American 17 (28.8 percent); Other or multiple 4 (6.8 percent).* Ethnicity: Hispanic or Latino 24 (40.7 percent); Not Hispanic or Latino 35 (59.3 percent).* Body mass index: mean (S D) of 29.9 (6.4) kg/m super 2.* Baseline T D severity: Mild (A I M S item 8 score = 2) 24 (40.7 percent); Moderate/severe (A I M S item 8 score = 3 or 4) 35 (59.3 percent).* Psychiatric diagnosis: Schizophrenia or schizoaffective disorder 27 (45.8 percent); Major depressive disorder or bipolar disorder 32 (54.2 percent).* Baseline P R O scores (n = 54): Tardive Dyskinesia Impact Scale total (range 0 to 44) 14.3 (8.1); Sheehan Disability Scale domains (range 0 to 10) including Work/school 3.1 (3.2), Social life 4.4 (2.8), and Family life 3.2 (3.0); E Q-Visual Analogue Scale (range 0 to 100) 66.1 (18.0).* Baseline Clin R O scores (n = 54): Abnormal Involuntary Movement Scale total (range 0 to 28) 11.1 (4.0); Clinical Global Impression of SeverityâTardive Dyskinesia (range 1 to 7) 3.9 (0.7).

Demographics:

* Age: mean 61.3 years, S D 12.0 .

* Sex: Female n equals 34 (57.6 percent), Male n equals 25 (42.4 percent).

* Race: White n equals 38 (64.4 percent), Black or African American n equals 17 (28.8 percent), Other or multiple n equals 4 (6.8 percent).

* Ethnicity: Hispanic or Latino n equals 24 (40.7 percent), Not Hispanic or Latino n equals 35 (59.3 percent).

* Body mass index: mean 29.9, S D 6.4 kilograms per meter squared.

Clinical Characteristics:

* Baseline T D severity: Mild (A IMS item 8 score equals 2) n equals 24 (40.7 percent), Moderate or severe (A IMS item 8 score equals 3 or 4) n equals 35 (59.3 percent).

* Psychiatric diagnosis: Schizophrenia or schizoaffective disorder n equals 27 (45.8 percent), Major depressive disorder or bipolar disorder n equals 32 (54.2 percent).

Mean Mean Baseline P R O scores:

* Tardive dyskinesia impact scale total (0 to 44): 14.3 (S D 8.1).

* Sheehan disability scale domains (0 to 10): Work or school 3.1 (S D 3.2), Social life or leisure activities 4.4 (S D 2.8), Family life or home responsibilities 3.2 (S D 3.0).

* E Q visual analogue scale (0 to 100): 66.1 (S D 18.0).

Baseline Clin R O scores (mean):

* Abnormal involuntary movement scale total (0 to 28): 11.1 (S D 4.0).

* Clinical global impression of severity tardive dyskinesia (1 to 7): 3.9 (S D 0.7).

Navigate back to Table 1..

### Figure 2. Long description

A vertical flowchart organized as follows:

* At the top, 119 Screened.

* Two lateral branches lead to exclusions:

- Left: 21 Met Exclusion Criteria, including hepatic impairment n equals 6, positive drug screen n equals 4, unstable medical condition n equals 4, prohibited medication n equals 3, Q T interval >450 milliseconds male or >470 milliseconds female n equals 2, B A R S Global score greater than or equal to 2 n equals 1, and risk of suicidal behavior n equals 1.

- Right: 39 Did Not Meet Inclusion Criteria, including insufficient T D movement severity n equals 25, unable to comply n equals 5, unstable psychiatric regimen n equals 3, lacked awareness of movements n equals 2, B M I <18 or >42.0 kilograms per meter squared n equals 1, no T D diagnosis n equals 1, and other n equals 2.

* The main path leads to 59 Enrolled.

* From Enrolled, a left branch points to Analysis Sets: Full analysis set n equals 54, Primary analysis set n equals 45, and Safety analysis set n equals 59.

* A right branch from the main path shows 7 Discontinued: Lost to follow up n equals 2, Adverse event n equals 1, Death n equals 1, Investigator decision n equals 1, Withdrawal of consent n equals 1, and Other n equals 1.

* The main path continues to 52 Completed Week 24 Visit.

* A final right branch shows 1 Discontinued due to Other.

* The flowchart ends at the bottom with 51 Completed Study.

Navigate back to Figure 2..

### Figure 3. Long description

A multi-panel figure containing five line graphs labeled A through E. Each graph plots Mean C F B with 95 percent C I on the Y-axis against time points at Baseline, Week 4, Week 8, Week 16, and Week 24 on the X-axis. A vertical arrow on the right of each graph indicates the direction of improvement.

Panel A. Tardive Dyskinesia Impact Scale Total Score. The line shows a downward trend from 0 at baseline to negative 8.0 at Week 24. A dashed horizontal line marks the M C I D at negative 4 points, which is surpassed by Week 8. Mean scores are 14.3 at baseline (S D 8.1) and 5.8 at Week 24 (S D 5.2).

Panel B. Sheehan Disability Scale Social and Leisure Score. The line trends downward from 0 to negative 2.3 at Week 24. Mean scores S D are 4.4 at baseline (S D 2.8) and 2.3 at Week 24 (S D 2.0).

Panel C. Sheehan Disability Scale Family and Home Score. The line trends downward from 0 to negative 1.6 at Week 24. Mean scores are 3.2 at baseline (S D 3.0) and 1.8 at Week 24 (S D 2.2).

Panel D. Sheehan Disability Scale Work and School Score. The line trends downward from 0 to negative 1.2 at Week 24. Mean scores are 3.1 at baseline (S D 3.2) and 1.6 at Week 24 (S D 2.5).

Panel E. E Q Visual Analogue Scale Score. Unlike the others, improvement is upward. The line increases from 0 at baseline to 13.1 at Week 24. Mean scores are 66.1 at baseline (S D 18.0) and 79.5 at Week 24 (S D 13.1).

Navigate back to Figure 3..

### Figure 4. Long description

Panel A. AIMS Total Score (Sum of Items 1 through 7). The y-axis shows Mean C F B (95 percent C I) from 0 to minus 10. The x-axis shows time points: Baseline (n equals 54), Week 4 (n equals 53), Week 8 (n equals 53), Week 16 (n equals 52), and Week 24 (n equals 45). A horizontal dashed line marks the M C I D at negative 2 points. The data points show a downward trend: negative 2.7 at Week 4, negative 4.3 at Week 8, negative 5.5 at Week 16, and negative 6.8 at Week 24. A vertical arrow on the right points down indicating improvement.

Panel B. C G I T D S Score. The y-axis shows Mean C F B (95 percent C I) from 0 to minus 3. The x-axis time points and sample sizes are identical to Panel A. The data points show a downward trend: negative 0.4 at Week 4, negative 0.8 at Week 8, negative 1.1 at Week 16, and negative 1.5 at Week 24. A vertical arrow on the right points down indicating improvement.

Panel C. P G I C Response. The y-axis shows Percentage of Participants from 0 to 80. The x-axis shows Week 4, Week 8, Week 16, and Week 24. Each bar is stacked with two categories: 1 (very much improved) in dark teal and 2 (much improved) in light teal.

* Week 4: 3.8 percent (very much improved), 17.0 percent (much improved), total 20.8 percent.

* Week 8: 13.2 percent (very much improved), 22.6 percent (much improved), total 35.8 percent.

* Week 16: 21.2 percent (very much improved), 36.5 percent (much improved), total 57.7 percent.

* Week 24: 28.9 percent (very much improved), 42.2 percent (much improved), total 71.1 percent.

Navigate back to Figure 4..

### Table 2. Long description

The table is organized into seven columns: Subgroup, Baseline n, Baseline Mean score (S D), Week 24 n, Week 24 Mean score (S D), Week 24 n (for change), and Week 24 Mean C F B [95% C I].Data is grouped by four metrics:1. T D I S total:- Mood: Baseline 15.9 (6.8); Week 24 5.1 (5.0); C F B -9.7 [-12.7, -6.7].- Psychotic: Baseline 12.4 (9.3); Week 24 6.7 (5.5); C F B -5.8 [-10.2, -1.4].- T D-Mild: Baseline 12.9 (8.0); Week 24 6.2 (4.7); C F B -6.8 [-10.7, -2.9].- T D-Mod/Severe: Baseline 15.3 (8.1); Week 24 5.6 (5.7); C F B -8.9 [-12.4, -5.4].2. S D S social/leisure:- Mood: Baseline 4.7 (2.5); Week 24 2.0 (1.6); C F B -2.9 [-4.1, -1.7].- Psychotic: Baseline 4.1 (3.1); Week 24 2.7 (2.4); C F B -1.6 [-3.0, -0.2].- T D-Mild: Baseline 3.9 (2.6); Week 24 2.2 (2.2); C F B -1.8 [-3.3, -0.2].- T D-Mod/Severe: Baseline 4.8 (2.9); Week 24 2.4 (1.9); C F B -2.8 [-3.9, -1.7].3. S D S family/home:- Mood: Baseline 3.8 (2.6); Week 24 1.6 (1.8); C F B -2.3 [-3.5, -1.0].- Psychotic: Baseline 2.5 (3.2); Week 24 2.2 (2.6); C F B -0.7 [-2.3, +1.0].- T D-Mild: Baseline 2.9 (2.8); Week 24 1.9 (2.1); C F B -1.3 [-3.0, +0.4].- T D-Mod/Severe: Baseline 3.4 (3.1); Week 24 1.8 (2.3); C F B -1.8 [-3.0, -0.5].4. E Q-VAS:- Mood: Baseline 62.6 (16.5); Week 24 80.1 (14.2); C F B +17.0 [+9.3, +24.7].- Psychotic: Baseline 70.4 (19.2); Week 24 78.8 (11.9); C F B +8.3 [+0.7, +15.8].- T D-Mild: Baseline 65.6 (15.7); Week 24 80.0 (12.4); C F B +12.8 [+4.0, +21.7].- T D-Mod/Severe: Baseline 66.4 (19.6); Week 24 79.1 (13.8); C F B +13.3 [+6.1, +20.6].

1. T D I S total (Tardive Dyskinesia Impact Scale):

- Mood: Baseline mean 15.9; Week 24 mean 5.1; Mean C F B -9.7 [95% C I: -12.7, -6.7].

- Psychotic: Baseline mean 12.4; Week 24 mean 6.7; Mean C F B -5.8 [95% C I: -10.2, -1.4].

- T D-mild: Baseline mean 12.9; Week 24 mean 6.2; Mean C F B -6.8 [95% C I: -10.7, -2.9].

- T D-mod/severe: Baseline mean 15.3; Week 24 mean 5.6; Mean C F B -8.9 [95% C I: -12.4, -5.4].

2. S D S social/leisure (Sheehan Disability Scale):

- Mood: Baseline mean 4.7; Week 24 mean 2.0; Mean C F B -2.9.

- Psychotic: Baseline mean 4.1; Week 24 mean 2.7; Mean C F B -1.6.

- T D-mild: Baseline mean 3.9; Week 24 mean 2.2; Mean C F B -1.8.

- T D-mod/severe: Baseline mean 4.8; Week 24 mean 2.4; Mean C F B -2.8.

3. S D S family/home:

- Mood: Baseline mean 3.8; Week 24 mean 1.6; Mean C F B -2.3.

- Psychotic: Baseline mean 2.5; Week 24 mean 2.2; Mean C F B -0.7.

- T D-mild: Baseline mean 2.9; Week 24 mean 1.9; Mean C F B -1.3.

- T D-mod/severe: Baseline mean 3.4; Week 24 mean 1.8; Mean C F B -1.8.

4. E Q - VAS (EuroQoL Visual Analogue Scale):

- Mood: Baseline mean 62.6; Week 24 mean 80.1; Mean C F B +17.0.

- Psychotic: Baseline mean 70.4; Week 24 mean 78.8; Mean C F B +8.3.

- T D-mild: Baseline mean 65.6; Week 24 mean 80.0; Mean C F B +12.8.

- T D-mod/severe: Baseline mean 66.4; Week 24 mean 79.1; Mean C F B +13.3.

Across all subgroups, T D I S and S D S scores decreased (indicating improvement), while E Q - VAS scores increased (indicating improved health status).

Navigate back to Table 2..

### Table 3. Long description

The table is divided into three main sections based on clinical scales. Each section includes four subgroups: Mood, Psychotic, T D dash Mild, and T D dash Mod forward slash Severe.1. A I M S total section:- Mood: Baseline mean 12.0 (n=30); Week 24 mean 4.0 (n=25); Mean C F B -7.6.- Psychotic: Baseline mean 10.0 (n=24); Week 24 mean 3.9 (n=20); Mean C F B -5.8.- T D dash Mild: Baseline mean 9.1 (n=22); Week 24 mean 3.4 (n=20); Mean C F B -5.6.- T D dash Mod forward slash Severe: Baseline mean 12.5 (n=32); Week 24 mean 4.4 (n=25); Mean C F B -7.8.2. C G I dash T D dash S section:- Mood: Baseline mean 4.1; Week 24 mean 2.6; Mean C F B -1.5.- Psychotic: Baseline mean 3.8; Week 24 mean 2.4; Mean C F B -1.4.- T D dash Mild: Baseline mean 3.5; Week 24 mean 2.3; Mean C F B -1.2.- T D dash Mod forward slash Severe: Baseline mean 4.3; Week 24 mean 2.7; Mean C F B -1.7.3. P G I dash C section (Week 24 only, showing score less than or equal to 2):- Mood: 19 of 25 participants (76.0%).- Psychotic: 13 of 20 participants (65.0%).- T D dash Mild: 14 of 20 participants (70.0%).- T D dash Mod forward slash Severe: 18 of 25 participants (72.0%).

1. A I M S total score section:

- Mood subgroup: Baseline mean 12.0 (n equals 30); Week 24 mean 4.0 (n equals 25); Mean C F B -7.6.

- Psychotic subgroup: Baseline mean 10.0 (n equals 24); Week 24 mean 3.9 (n equals 20); Mean C F B -5.8.

- T D mild subgroup: Baseline mean 9.1 (n equals 22); Week 24 mean 3.4 (n equals 20); Mean C F B -5.6.

- T D mod severe subgroup: Baseline mean 12.5 (n equals 32); Week 24 mean 4.4 (n equals 25); Mean C F B -7.8.

2. C G I T D S score section:

- Mood subgroup: Baseline mean 4.1; Week 24 mean 2.6; Mean C F B -1.5.

- Psychotic subgroup: Baseline mean 3.8; Week 24 mean 2.4; Mean C F B -1.4.

- T D mild subgroup: Baseline mean 3.5; Week 24 mean 2.3; Mean C F B -1.2.

- T D mod severe subgroup: Baseline mean 4.3; Week 24 mean 2.7; Mean C F B -1.7.

3. P G I C section (Week 24 only, showing percentage with score less than or equal to 2):

- Mood: 19 participants (76.0 percent).

- Psychotic: 13 participants (65.0 percent).

- T D mild: 14 participants (70.0 percent).

- T D mod severe: 18 participants (72.0 percent).

Navigate back to Table 3..

### Table 4. Long description

The table consists of two columns. The first column lists the event category and the second column provides the count n and percentage for all participants where N equals 59.

Summary statistics:

* Any T E A E: n equals 28 (47.5percent)

* Any T E A E leading to a dose reduction: n equals 2 (3.4percent)

* Any T E A E leading to study drug discontinuation: n equals 2 (3.4percent)

* Any serious T E A E: n equals 6 (10.2percent)

* Any T E A E resulting in death: n equals 1 (1.7percent)

Common T E A Es reported in 2 percent or more of participants:

* Nausea: n equals 3 (5.1percent)

* Somnolence: n equals 3 (5.1percent)

* Suicidal ideation: n equals 3 (5.1percent)

* Depression: n equals 2 (3.4percent)

* Headache: n equals 2 (3.4percent)

* Insomnia: n equals 2 (3.4percent)

* Tremor: n equals 2 (3.4percent)

* Vomiting: n equals 2 (3.4percent)

Navigate back to Table 4..
