# Supplementary material for: Valbenazine improves quality of life, functionality, and movement severity in patients with tardive dyskinesia: findings from the KINECT-PRO™ study
Source: CNS Spectr. 2026 Aug 11;31(1):e30. doi: 10.1017/S1092852926101138 (PMC13732611; doi:10.1017/S1092852926101138)
Supplement: Supplementary file 1 [file S1092852926101138sup001.pdf]

SUPPLEMENTARY MATERIALS

Table S1 Key eligibility criteria

**Inclusion Criteria**
• Provide informed consent
• At least 18 years of age
• Diagnosis of schizophrenia or schizoaffective disorder, bipolar disorder, or MDD for ≥3 months prior to screening (per DSM-5)
• Diagnosis of neuroleptic-induced TD for ≥3 months prior to screening (per DSM-5)
• At least mild TD, defined by an AIMS item 8 score of ≥2 at screening
• Have at least moderate dyskinetic movements in ≥1 body area (AIMS score ≥3) or at least mild dyskinetic movements in ≥2 body areas (AIMS score ≥2) at screening
• Have awareness of abnormal movements and at least mild distress associated with them as defined by an AIMS item 10 score of ≥2 at screening and baseline
• Stable psychiatric status per investigator judgment
• BMI of 18.0 to 42.0 kg/m^2, inclusive[a]
• Negative pregnancy tests at screening and baseline for females of childbearing potential
• Agreement to use effective contraception consistently from screening until 30 days after last dose of study treatment or final study visit
• Ability and willingness to comply with all study procedures, per investigator judgment
**Exclusion Criteria**
• Comorbid parkinsonism as assessed by the investigator, or mSAS score of >6 (excluding items 8 and 10) at screening or baseline
• Comorbid abnormal involuntary movements (e.g., parkinsonism, akathisia) more prominent than TD
• BARS global clinical assessment score ≥2 at screening or baseline
• BPRS total score of ≥50 at screening or at baseline
• Significant risk of suicidal or violent behavior during the 3 months prior to screening or at baseline
• Diagnosis of moderate or severe substance use disorder within 6 months before screening per the MINI
• Positive urine drug screen for disallowed substances (e.g., amphetamines, cocaine, methadone, methamphetamine, MDMA, phencyclidine, and nonprescribed benzodiazepines, opiates, or barbiturates)<br>• Cannabinoids allowed if participant did not test positive for a moderate or severe substance use disorder (per MINI); legal marijuana use (per local laws) limited to ≤3 times per week
• Active, clinically significant unstable medical condition within 30 days of baseline<br>• Any laboratory value outside the normal range that was clinically significant per investigator judgment
• History of severe hepatic impairment or elevation of any of the following laboratory tests: serum creatinine >1.5 × ULN, serum AST ≥3.0 × ULN, serum ALT ≥3.0 × ULN, GGT ≥3.0 × ULN, serum total bilirubin >2.0 × ULN (participants with Gilbert syndrome not required to meet bilirubin criteria)
• History of long QT syndrome or cardiac arrhythmia, including uncontrolled bradyarrhythmia and heart failure
• Triplicate ECG QTcF of >450 msec (male participants) or >470 msec (female participants) or the presence of any clinically significant cardiac abnormality during screening
• Hematologic malignancy or solid tumor diagnosed within 3 years prior to screening or not in remission
• History of neuroleptic malignant syndrome
• Use of any active investigational drug from a clinical study within 30 days or 5 half-lives before screening (whichever is longer), or participation in ≥3 clinical studies within 12 months before screening
• Prior (within 1 month of screening) or concomitant use of any VMAT2 inhibitor<br>• History of intolerance to VMAT2 inhibitors or hypersensitivity to any component of the valbenazine formulation

[a]Amended from the original value of 18 to 40.5 kg/m^2.

AIMS, Abnormal Involuntary Movement Scale; ALT, serum alanine aminotransferase; AST, serum aspartate aminotransferase; BARS, Barnes Akathisia Rating Scale; BMI, body mass index; DSM-5, *Diagnostic and Statistical Manual of Mental Disorders*, 5th edition; ECG, electrocardiogram; GGT, gamma-glutamyl transferase; MDD, major depressive disorder; MDMA, 3,4-methylenedioxymethamphetamine; MINI, Mini International Neuropsychiatric Interview; mSAS, modified Simpson Angus Scale; QTcF, corrected QT interval using Fridericia's formula; TD, tardive dyskinesia; ULN, upper limit of normal; VMAT2, vesicular monoamine transporter 2.

Table S2 Allowed and prohibited medications

**Allowed Medications**
• Benzodiazepines (at equivalent of ≤3 mg/d lorazepam)
• Medication(s) for schizophrenia, schizoaffective disorder, bipolar disorder, or MDD (if on stable regimen for 30 days prior to baseline)<br>▪ Including (but not limited to) antipsychotics, selective serotonin reuptake inhibitors, and serotonin-norepinephrine reuptake inhibitors
• Barbiturates / opiates (if on stable dose for at least 2 weeks prior to baseline)
**Prohibited Medications**
• Antiemetics (e.g., metoclopramide, prochlorperazine, promethazine)
• Botulinum toxin (for 90 days prior to screening)
• CYP3A4 strong inducers (e.g., phenytoin, phenobarbital, rifabutin, rifampin, primidone, St. John's Wort, carbamazepine)
• Dopamine agonists and precursors (e.g., ropinirole, pramipexole, carbidopa/levodopa)
• MAOIs (e.g., isocarboxazid, phenelzine, selegiline, tranylcypromine)
• Stimulants (e.g., amphetamine, methylphenidate, ephedrine)
• VMAT2 inhibitors (e.g., tetrabenazine, deutetrabenazine) or reserpine

CYP, cytochrome P450; MAOI, monoamine oxidase inhibitors; VMAT2, vesicular monoamine transporter 2.

Figure S1 Valbenazine dosing through Week 24 (FAS)

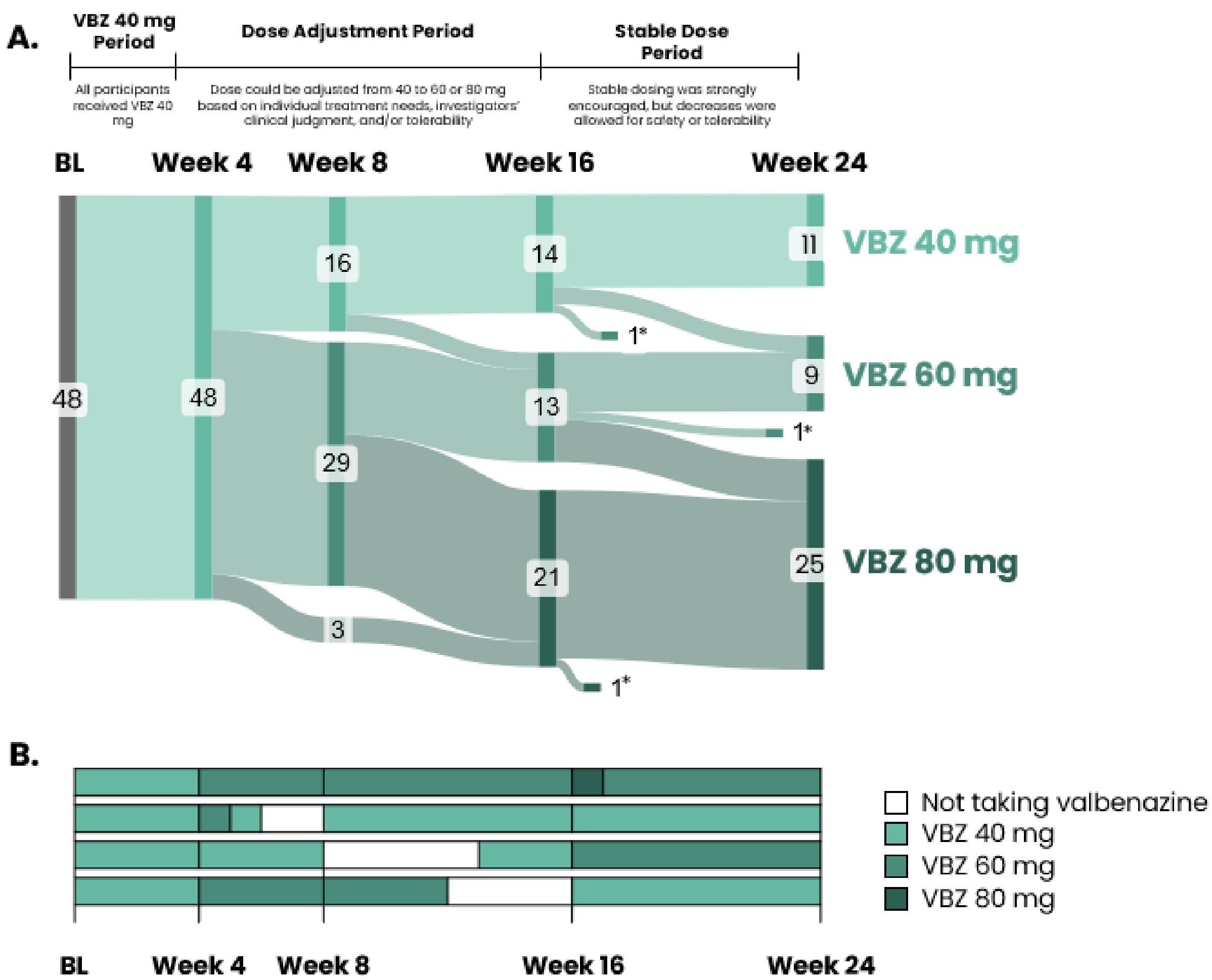

The Sankey diagram **(A)** includes 3 participants (*) who stopped treatment before Week 24: 1 who had been taking 80 mg from Weeks 8 to 16; 1 who had been taking 60 mg from Weeks 8 to 16 (who was included in Week 24 analyses); and 1 who was escalated from 40 to 60 mg at Week 16. It excludes 2 participants whose stop date of treatment are unknown.
The swimmer plot **(B)** shows dose changes for 4 participants with more complicated trajectories. These included: 1 participant who took valbenazine 60 mg continuously from Week 4 through Week 24, with a brief (~1 week) escalation to 80 mg; and 3 participants who temporarily stopped treatment during the study but were taking valbenazine 40 mg (n=2) or 60 mg (n=1) at Week 24.

BL, baseline; FAS, full analysis set; TEAE, treatment-emergent adverse event; VBZ, valbenazine.

Table S3 Safety assessments (safety analysis set)

Safety Assessment	At Baseline		At Week 24	
	n	Mean Value (SD)	n	Mean CFB (SD)
**Laboratory tests**				
Alanine aminotransferase, U/L	59	21.9 (14.3)	48	-2.4 (11.8)
Aspartate aminotransferase, U/L	59	21.5 (11.3)	48	-1.2 (10.6)
Total bilirubin, mg/dL	59	0.3 (0.2)	48	-0.02 (0.1)
Blood urea nitrogen, mg/dL	59	15.8 (7.5)	48	-0.7 (4.8)
Creatine kinase, U/L	59	155.5 (196.0)	48	-2.0 (81.5)
**Vital signs**				
Supine systolic blood pressure, mmHg	59	127.1 (12.0)	47	+1.9 (10.1)
Supine diastolic blood pressure, mmHg	59	77.6 (7.8)	47	+0.2 (9.2)
Orthostatic systolic blood pressure, mmHg[a]	59	0.6 (7.9)	46	-0.5 (7.6)
Orthostatic diastolic blood pressure, mmHg[a]	59	1.9 (5.6)	46	-0.3 (4.9)
Supine pulse rate, bpm	59	76.2 (10.9)	47	-3.1 (12.0)
**Electrocardiogram**				
PR interval, ms	59	167.9 (21.4)	48	-0.6 (14.1)
QRS duration, ms	59	93.5 (13.9)	48	-0.6 (4.6)
QTcF interval, ms	59	414.7 (21.0)	46	+1.3 (13.6)
**Movement scales**				
BARS total score[b]	56	0.3 (0.5)	47	-0.1 (0.7)
BARS global score[c]	56	0.0 (0.2)	47	0.0 (0.3)
Modified SAS total score[d]	56	2.3 (2.6)	47	-0.7 (1.4)

[a]Calculated as standing value minus supine value.

[b]Score range: 0 to 9, with higher scores indicating worse severity.

[c]Score range: 0 (absent) to 5 (severe akathisia).

[d]Score range: 0 to 40, with higher scores indicating worse severity.

BARS, Barnes Akathisia Rating Scale; CFB, change from baseline; QTcF, corrected QT interval using Fridericia's formula; SAS, Simpson-Angus Scale; SD, standard deviation.
